# We Treat Them Better Than the Family They Have: Negotiating Aide-Family Relationships in the Home Care Setting

**DOI:** 10.1093/geroni/igaa057.1895

**Published:** 2020-12-16

**Authors:** Emily Franzosa, Emma Tsui

**Affiliations:** 1 James J. Peters VA Medical Center, Brooklyn, New York, United States; 2 CUNY Graduate School of Public Health & Health Policy, New York, New York, United States

## Abstract

Unpaid and paid care in the home are closely intertwined, but a lack of outside supervision and support often forces family and non-family caregivers to negotiate care tasks and boundaries alone, leading to role conflict and role ambiguity. This analysis drew on two qualitative studies of home health aides (S1 n = 27, S2 n =26) to explore 1) aides’ perception of their caretaking role; 2) aides’ experiences co-producing care with family members; and 3) factors affecting these relationships. Data were analyzed through grounded theory and discourse analysis. We identified three relationship dynamics between aides and family members: independent, where aides and families provided care separately; competitive, where aides and families struggled over control of care tasks; and carative, where aides considered family part of the unit of care. We propose strategies for employer agencies to better support paid and unpaid caregivers in negotiating boundaries and co-producing care.

